# Reprogramming of Metabolic and Adipokine-related Gene Expression in 3D Mammary Epithelial Organoids by Dietary Bioactive Compounds

**DOI:** 10.1007/s10911-026-09612-z

**Published:** 2026-07-29

**Authors:** Mariona Palou, Marta Alonso-Bernáldez, Andreu Palou-March, Andreu Palou, Catalina Picó, Francisca Serra

**Affiliations:** 1 Group of Nutrigenomics, Biomarkers and Risk Evaluation (NuBE), University of the Balearic Islands, Palma, Spain; 2 Institut d’Investigació Sanitària Illes Balears (IdISBa), Palma, Spain; 3 CIBER de Fisiopatología de la Obesidad y Nutrición (CIBEROBN), Instituto de Salud Carlos III, Madrid, Spain; 4 Artificial Intelligence Research Institute of the Balearic Islands (IAIB), Palma, Spain; 5 Alimentómica S.L. (Spin Off No. 001 From UIB), Palma, Spain

**Keywords:** Oleic Acid, Betaine, Leucine, Leptin, Adiponectin, Mammary Epithelial Organoids

## Abstract

Dietary bioactive compounds can modulate mammary gland gene expression, potentially influencing milk composition and infant metabolic programming. This study investigated whether selected dietary bioactive compounds modulate leptin and adiponectin expression in rat mammary epithelial organoids (MEO), a 3D in vitro model of the mammary gland. 18 nutrients were initially screened for effects on leptin gene expression, followed by confirmatory and dose-dependent analyses focusing on oleic acid (OA), leucine (Leu), betaine (Bet), and their triple combination. mRNA levels of leptin, adiponectin, and β-casein (*Csn2*), as well as selected genes related to one-carbon and lipid metabolism and specific microRNAs, were analyzed. OA, alone or in combination, consistently increased leptin, adiponectin, and *Csn2* mRNA expression in MEO compared with controls. These effects were accompanied by downregulation of lipogenic markers, including *Srebp1*, and increased *Cpt1b* expression under the triple-combination treatment, without significant changes in miRNA levels. In contrast, Bet and Leu showed more variable, context-dependent effects on leptin, adiponectin, and *Csn2* expression, while altering miRNA profiles that differed from those observed under OA-containing treatments. Overall, the results indicate that dietary bioactive compounds modulate gene expression of rat MEO.

**Supplementary Information:**

The online version contains supplementary material available at https://doi.org/10.1007/s10911-026-09612-z.

## Introduction

Human breast milk is a highly complex and dynamic fluid, biologically tailored to provide newborns with the essential nutrients and bioactive compounds required for optimal postnatal development. Beyond its primary role as a source of energy and structural components, breast milk functions as a multifaceted bioactive system. Its matrix contains a blend of macro- and micronutrients, and a diverse array of bioactive compounds, growth and immunological factors, and microorganisms [1, 2]. Milk components are either synthesized by the mammary epithelial cells (MECs) within the mammary gland (MG) or transferred from maternal circulation, reflecting both the mother's endogenous stores and her recent dietary intake [2]. This intrinsically complex composition exerts profound influence on the neonate’s physiology and development, with potential long-term implications for metabolic health.

Among the hormones present in breast milk, leptin plays an important role. It can be absorbed across the immature gastric mucosa of the neonate and represents a major source of leptin during the early postnatal period [3–5]. Evidence from animal studies, supported by observational data in humans, suggests that ingested leptin during lactation exerts a metabolic programming effect on long-term body weight regulation and metabolic health [6–8]. Notably, leptin concentrations in maternal circulation and breast milk exhibit significant plasticity. They vary according to maternal adiposity and are also influenced by dietary patterns and specific nutrients [9–11].

Adiponectin is another key adipokine present in breast milk, typically at concentrations substantially higher (40-fold greater) than those of other adipokines [12, 13]. Maternal diet can also modulate milk adiponectin levels [14–16]. Although its effects on infant growth and metabolic outcomes remain unclear, this hormone has been implicated in the regulation of energy metabolism and insulin sensitivity during early life [15–17]. In previous studies using rat models, we found that maternal supplementation during lactation with a combination of oleic acid (OA), betaine (Bet), and leucine (Leu) altered milk composition [16]. Specifically, this supplementation increased leptin and decreased adiponectin concentrations and modified the milk miRNA profile, changes that were associated with alterations in offspring phenotype [16]. These findings prompted us to investigate whether such effects could result from direct actions of these nutrients on mammary epithelial cells. Therefore, identifying dietary compounds that regulate mammary production of leptin and adiponectin may help improve our understanding of nutritional regulation of milk composition.

Despite these associations, the mechanisms by which specific nutrients modulate adipokine synthesis and secretion in the mammary gland remain poorly elucidated. Addressing this question requires the use of controlled experimental models. In this context, primary mammary epithelial organoid (MEO) 3D cultures provide a physiologically relevant platform for studying nutrient-induced changes in mammary gland gene expression and metabolic pathways under controlled conditions and may complement findings from in vivo studies [18].

The present study evaluated the effects of selected nutrients on leptin and adiponectin gene expression and associated metabolic signaling pathways using primary rat MEO cultures, a controlled in vitro model for the mammary gland. For this end, a panel of 18 bioactive compounds, commonly found in foods or dietary supplements, was selected for screening based on previous evidence of biological activity and classified into four groups: fatty acids; essential and non-essential amino acids; methionine cycle intermediates and methyl donors; and amino sugars and glycosaminoglycans.

## Materials and Methods

### Compound Selection

Compound selection was based on a targeted literature search in PubMed. Candidate nutrients, food-derived ingredients, and bioactive compounds were prioritized based on previous evidence reporting: (i) modulation of leptin expression or circulating leptin levels; (ii) effects on adiponectin expression or related metabolic signaling pathways; and/or (iii) associations with metabolic disease risk, including obesity, type 2 diabetes, and cardiovascular disorders. Additional criteria included evidence of biological relevance during pregnancy and lactation, such as the reported presence of the compound in breast milk.

The literature review also considered studies reporting circulating levels of these compounds in humans or animals, as well as in vitro or in vivo experimental interventions, in order to define physiologically relevant concentrations for subsequent in vitro testing.

Based on these criteria, a panel of 18 bioactive compounds was selected for in vitro screening (Study 1). A detailed rationale and supporting references are provided in Supplementary Table S1.

### Primary Culture of Rat Mammary Epithelial Organoids

Primary mammary epithelial organoid (MEO) cultures were established using a protocol adapted from Hahm and Ip [19]. For each 24-well plate, MG were collected from two virgin female Wistar rats aged 38–42 days. Nonadherent organoids were resuspended in 1250 µL of Matrigel® Matrix (Corning, REF 356234) and seeded into a TC-treated BioCoat™ Matrigel® Matrix 24-well plates (Corning, REF 354433) with 50 µL per well. Prior to seeding, plates were rehydrated with 350 µL of DMEM:F12 (Gibco, Thermo Fisher, REF 11320033). After seeded, plates were incubated at 37ºC, 5% CO_2_ for 30 min to allow Matrigel® Matrix polymerization. Cultures were maintained for 20 days to achieve MEO differentiation. The culture medium consisted of DMEM:F12 supplemented with the following hormones and growth factors: insulin (10 µg/mL), progesterone (1 µg/mL), hydrocortisone (1 µg/mL), apo-transferrin (5 µg/mL), ascorbic acid (5 mM), EGF (10 ng/mL), gentamicin (50 µg/mL), BSA (1 mg/mL), and prolactin (1 µg/mL). A total volume of 500 µL of complete medium was added per well.

All experimental treatments were prepared and diluted in absolute ethanol or sterile cell culture water as the respective vehicles. A subset of wells in each plate received only the corresponding vehicles, serving as the negative control group. The final concentration of vehicle (water and/or ethanol) was kept constant across all treatment and control wells. The data presented were derived from four independent cultures. Each independent culture consisted of organoids initiated from the mammary tissue of a distinct animal. Cultures were established on separate days using independent preparations. In all experiments, cells were harvested on day 20 of culture.

#### Study 1. Screening of Bioactive Compounds and Culture Characterization

A panel of 18 bioactive compounds was tested at two physiological doses for 24 h on day 19 of culture, with six replicate wells per treatment. The complete list of compounds and concentrations is provided in Table 1. Leptin mRNA expression was analyzed by qPCR. Based on the screening results, a subset of compounds showing the strongest effects on leptin expression was selected for further analysis. This included five compounds tested at both doses and three additional compounds tested at the lower dose only. For these compounds, leptin expression was re-evaluated and gene expression analysis of MG differentiation-related markers was performed to explore potential mechanisms. The selected compounds for validation and mechanistic analysis are indicated in Table 1.

**Table 1:** List of all the Bioactive Components Tested in MEO at the Lowest and Highest Doses in Study 1

Treatment for MEO		
**Bioactive component**	**Lowest dose**	**Highest dose**
Oleic acid (OA)	200 μM *	1000 μM
Linoleic acid (LA)	250 μM	1250 μM
Alpha-Linolenic acid (ALA)	6 μM *	30 μM
cis-9, trans-11-conjugated linoleic acid (CLA-9,11)	20 μM	100 μM
cis-5,8,11,14,17-Eicosapentaenoic acid (EPA)	15 μM	75 μM
cis-4,7,10,13,16,19-Docosahexaenoic acid (DHA)	15 μM	75 μM
Propanoic acid (PA) (or propionic acid)	10 μM	50 μM *
Butyric acid	10 μM	50 μM
L-Leucine (Leu)	125 μM *	625 μM *
L-Methionine	50 μM	250 μM
L-Homocysteine (Hcy)	10 μM *	50 μM *
Choline	20 μM	100 μM
Betaine (Bet)	20 μM *	100 μM *
Folic acid	0.05 μM	0.25 μM
D-Glucosamine	1.5 mM	7.5 mM
N-acetyl-glucosamine (NAG)	1.5 mM	7.5 mM *
Hyaluronic acid (HA)	2.4 mg/L *	12 mg/L *
L-Glutamine	600 μM	3000 μM

^*^Indicate those treatments for which a second qPCR for leptin gene expression was done to validate the first preliminary results (Fig. 1)

**Fig. 1:**
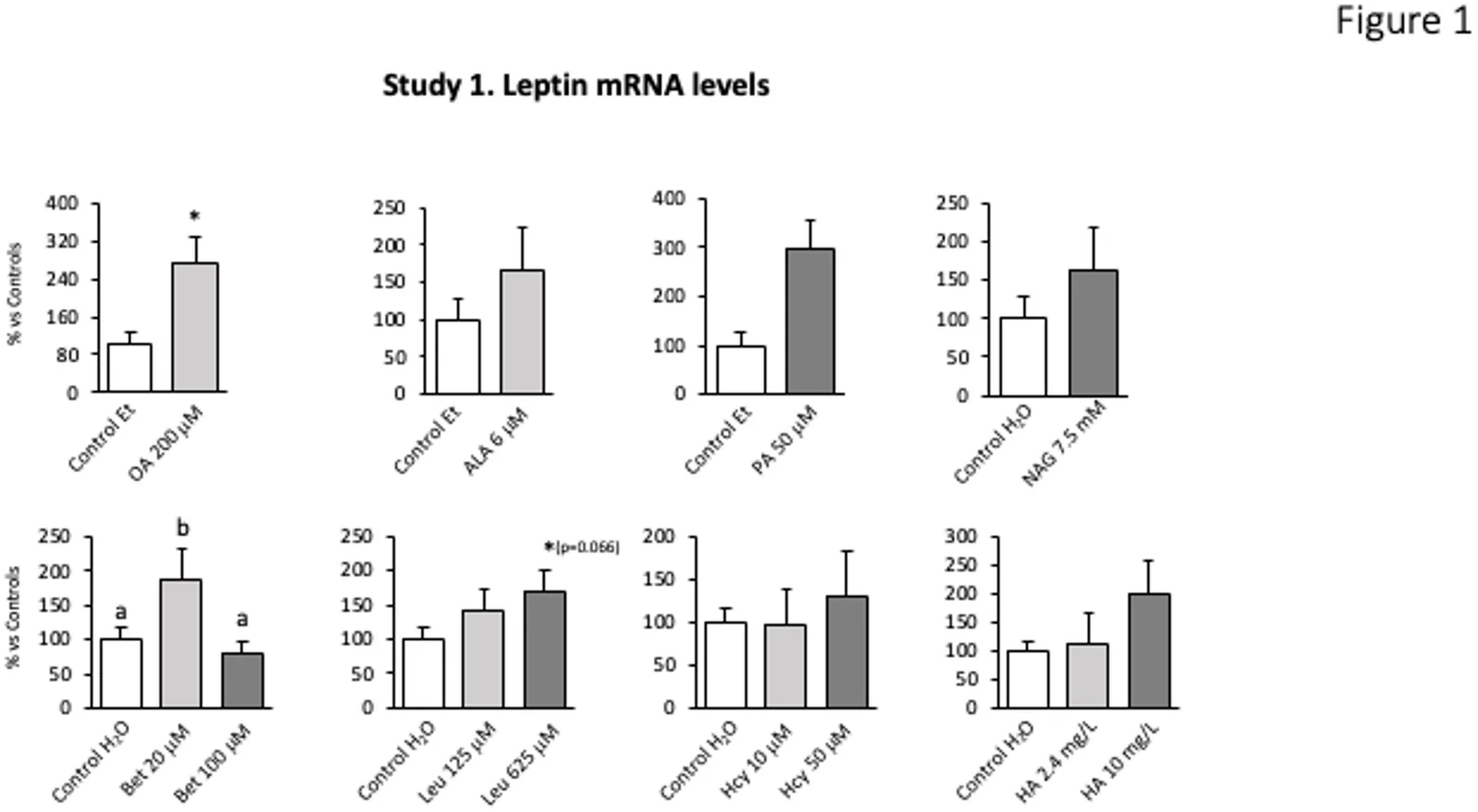
Study 1. mRNA Expression of Leptin in Rat MEOs Treated With Eight Selected Bioactive Compounds At Different Doses. Data are Mean ± S.E.M. (*n* = 5–6) and are Expressed Relative to the Corresponding Control for Each Compound. Statistics: Student’s t-test Significant Differences: * vs Control. Threshold Significance was Set at *p* ≤ 0.05. Acronyms: Et, ethanol; OA, oleic acid; ALA, Alfa-linolenic Acid; PA, Propionic Acid; NAG, n-acetylglucosamine; Bet, Betaine; Leu, Leucine; Hcy, Homocysteine; and HA, Hyaluronic Acid

#### Study 2. Validation of the Effects of Selected Compounds from Study 1 on Leptin Gene Expression

Three independent MEO cultures were performed. In each culture, the three selected bioactive compounds from Study 1 (Bet, Leu, and OA) were tested individually at two doses, except OA, which was tested only at the lower dose based on the previous screening. Each condition included 8–15 replicate wells, with vehicle-treated wells serving as controls. All treatments were applied for 24 h on day 19 of the culture. Treatments were prepared by dilution in ethanol or water, as appropriate, following the procedures described above.

#### Study 3. Investigation of the Combinatorial Effects of the Selected Compounds Validated in Study 2

Three bioactive compounds validated in Study 2—Bet at 100 µM, Leu at 125 µM, and OA at 200 µM—were selected for combinatorial treatment analysis.

##### Acute Treatment

Each group of wells (individual compounds, dual combinations, and the triple combination) received acute treatment for 24 h on day 19 of culture, with 7–15 replicated wells per group.

##### Mid-term Treatment (7 Days)

A subset of wells was treated with the triple combination of OA, Leu, and Bet (designated as “Triple X”) for the final 7 days of culture. This prolonged exposure was included because MG cell cultures are generally considered differentiated from day 12–15 of culture onwards [19]. Four replicate wells were included, and the treatment medium was refreshed in parallel with the routine twice-weekly medium changes.

#### Study 4. Molecular Characterization of Gene and miRNA Expression Following Mid-term Treatment

A 7-day treatment was used to model sustained exposure based on Study 3 results, and gene and miRNA expression were subsequently analyzed under these settings. This experimental design focused on the three individual compounds (at a single dose) and the Triple X combination, guided by two main considerations: a) *Physiological relevance*: dietary compounds and nutritional interventions that modulate human milk composition are typically consumed over mid- to long-term periods rather than as acute doses. b) *Empirical data:* findings from Study 3 indicated that mid-term treatment with the Triple X combination produced a significantly greater increase in leptin gene expression compared to the 24-h acute treatment. This observation strongly suggested that prolonged exposure is required to fully elicit the molecular response.

### RNA Extraction

Total RNA was extracted from MEO using Tripure Reagent (Roche Diagnostic GmbH, Mannheim, Germany), according to the manufacturer’s instructions. RNA quantity was measured with the NanoDrop® ND-1000 spectrophotometer (Thermo Fisher), and its integrity was verified by agarose gel electrophoresis.

### mRNA Expression Analysis

Across all four experimental studies, modulation of leptin mRNA expression was used as the primary biological readout and was quantified for each treatment condition. In addition, to provide a broader mechanistic context, a general characterization of the gene expression level of key markers related to MG differentiation [20] was performed in Study 1. The genes analyzed were beta-casein (*Csn2*), alpha-casein 1 (*Csn1s1*), alpha-casein 2 (*Csn1s2a*), kappa-casein (*Csn3*), lactoalbumin (*Lalba*), prolactin receptor (*Prlr*). The expression of adiponectin (*Adipoq*) was also determined in Study 1 and 4, given its critical role in perinatal programming and its previously established reciprocal relationship with leptin in the context of milk composition.

For Study 4, mRNA levels of leptin, adiponectin, *Csn2*, selected lipid metabolism-related genes, and one-carbon (1C) metabolism-related genes were analyzed to characterize the impact of the treatments. Therefore, in addition to the above reported genes − *Lep*, *Adipoq* and *Csn2* −, the following were also determined: fat mass and obesity-associated protein (*Fto*), peroxisome proliferator-activated receptor alpha (*Ppara*), carnitine palmitoyltransferase 1 A (*Cpt1a*) and 1B (*Cpt1b*), peroxisome proliferator-activated receptor γ co-activator 1 alpha (*Pgc1a*), sterol regulatory element binding protein 1 (*Srebp1*), fatty acid synthase (*Fasn*), stearoyl-CoA desaturase (*Scd1*), solute carrier family 44 member 1 (*Slc44a1*), aldehyde dehydrogenase 7 family member A1 (*Aldh7a1*), betaine-homocysteine S-methyltransferase (*Bhmt*), sarcosine dehydrogenase (*Sardh*), serine hydroxymethyltransferase (*Shmt*), methylenetetrahydrofolate reductase (Mthfr), methyltetrahydrofolate-homocysteine methyltransferase (*Mtr*) and methionine adenosyltransferase 1a (*Mat1a*). Furthermore, the expression levels of genes selected as putative functional targets for the analyzed miRNAs were determined in Study 4. The selection process was based on high-confidence scores derived from recognized online databases for miRNA target prediction (mirDB, https://mirdb.org/; and TargetScan, https://www.targetscan.org/vert_80/). The genes studied were: cAMP-binding protein (*Crebp*), a target of miR-26a and 200b; insulin-induced gene 1 (*Insig1*), a target of miR-29a; peroxisome proliferator-activated receptor gamma (*Pparg*), a target of miR-27a; and phosphatase and tensin homolog (*Pten*), a target of miR-26a, miR-200a, miR-200b, and miR-221.

For the mRNA expression analyses, retrotranscription of total RNA was performed with the commercial iScript™ cDNA Synthesis kit (Bio-Rad, California, USA) following the manufacturer’s instructions. Each PCR was performed from diluted 1/5 cDNA template, forward and reverse primers (5 µM each), and Power SYBR Green PCR Master Mix (Applied Biosystems, Foster City, CA). Real-time qPCR was carried out in the StepOnePlus™ instrument under the following conditions: 10 min at 95 °C, followed by 40 cycles of 15 s at 95 °C and 1 min at 60 °C. For more restrictive conditions to analyze *Lep* mRNA levels (Study 1), the PCR conditions were: 7 min at 95 °C, followed by 50 cycles of 15 s at 95 °C, 20 s at 60 °C, and 40 s at 72 °C. Afterwards, a melting curve was generated for each reaction to verify the purity of the PCR products. The threshold cycle (Ct) was calculated by the instrument's software (StepOne Software v2.2.2, Applied Biosystems), and mRNA expression was calculated as a percentage of the control group using the 2^−ΔΔCt^ method (Livak and Schmittgen, 2001), with the average expression of beta-actin (*β-actin*) and GDP dissociation inhibitor 1 (*Gdi*) housekeeping genes as reference. All primers were obtained from Sigma and are detailed in Table 2.

**Table 2:** Primer Sequences Used for RT-qPCR Analysis

Gene ID	Forward	Reverse	Amplicon (bp)
*Lep*	TTCACACACGCAGTCGGTAT	AGGTCTCGCAGGTTCTCCAG	186
*Adipoq*	GCTCAGGATGCTACTGTTG	TCTCACCCTTAGGACCAAG	247
*Csn2*	ATCCTTGCCTGCCTTGTG	TGTTTTGTGGAATGGGACTG	250
*Fto*	CAGCAGTGGCAGCTGAAATA	AATGAGGATGCGAGATACCG	180
*Ppara*	TGTCGAATATGTGGGGACAA	AAACGGATTGCATTGTGTGA	215
*Cpt1b*	GCAAACTGGACCGAGAAGAG	CCTTGAAGAAGCGACCTTTG	180
*Cpt1a*	GCTCGCACATTACAAGGACAT	TGGACACCACATAGAGGCAG	250
*Pgc1a*	AGGAGGGTCATCGTTTGTGG	GGAGGCAGAAGAGCCGTC	256
*Srebp1*	CCCACCCCCTTACACACC	GCCTGCGGTCTTCATTGT	198
*Fasn*	CGGCGAGTCTATGCCACTAT	ACACAGGGACCGAGTAATGC	222
*Scd1*	ATCCCCTCCTCCAAGGTCTA	CGGGCCCATTCATATACATC	188
*Slc44a1*	TGCCTTTCACACCTATTCTGG	AGCGTTTTCCTTCCCTTTG	152
*Aldh7a1*	TTCTTCCCTCCGCTCTGTT	CGTGCCTCCTTCTTTCTTTG	242
*Bhmt*	AAGGAGGGTCTGGAAGCA	CTGATGTGGTAGGGCTCAAA	230
*Sardh*	CTTGTCTGTGGGTGGCTATG	GCTTATGGTCGGGTGTGAA	200
*Shmt*	AACCACTACAACCCACAAGACC	CCACAGCAACACCAGCAA	191
*Mthfr*	CAAGATGTGGGGTGAGGAG	TGGGAGTTGATGGTGAGGA	201
*Mtr*	GTCACCAGCGTGTCTCATTC	CCACAGCATCCACCAACTAC	242
*Mat1a*	GGGTCTGATGTTCGGCTATG	TCCAGTGTTATGTCTTCGTTGTG	243
*Crebp*	CAGGGAGGAGCAATACAGC	GGAGGACGCCATAACAACT	255
*Insig1*	ATCACCATCGCCTTCCTAGC	TGTTTCCCACTGTGACACCT	146
*Pparg*	GATCCTCCTGTTGACCCAGA	TCAAAGGAATGGGAGTGGTC	164
*Pten*	CAGAAAAAGTGGAAAATGGAAG	TGGAGAGAAGTATCGGTTGG	116
*β-actin*	ACGGGCATTGTGATGGACTC	GTGGTGGTGAAGCTGTAGCC	160
*Gdi1*	CCGCACAAGGCAAATACATC	GACTCTCTGAACCGTCATCAA	159

### miRNA Expression Analysis

In study 4, selected miRNAs were analyzed in parallel with gene expression. The miRNAs were chosen based on previous evidence [15–17] demonstrating their involvement in maternal metabolic status and their established correlation with leptin and adiponectin concentrations in milk. miRNA expression analysis was performed to assess potential regulatory changes associated with the treatments. Expression was quantified using previously described methods [16]. In short, TaqMan® MicroRNA Assays (Applied Biosystems) were used according to the manufacturer’s protocol. This method relies on a two-step of RT-PCR with probes. In the RT step, cDNA is obtained from total RNA samples using miRNA primers from the TaqMan MicroRNA Assays and reagents from the TaqMan® MicroRNA Reverse Transcription kit. RT was carried out under the following conditions: 30 min at 16 °C, 30 min at 42 °C, and 5 min at 85 °C. Real-time qPCR was carried out in the StepOnePlus™ instrument under the following conditions: 10 min at 95 °C, followed by 45 cycles of 15 s at 95 °C and 1 min at 60 °C. The threshold cycle (Ct) was calculated by the instrument's software (StepOne Software v2.2.2, Applied Biosystems), and relative miRNA expression was calculated by the 2^−ΔΔCt^ method (Livak and Schmittgen, 2001), with the average expression of U6 snRNA and beta-actin (β-actin) as reference. The following probes supplied from Applied Biosystems were used to detect specific miRNA: U6 snRNA as housekeeping small RNA (Assay ID: 001973), miR-26a (Assay ID: 000405), miR-27a (Assay ID: 000408), miR-29a (Assay ID: 002112), miR-103 (Assay ID: 000439), miR-200a (Assay ID: 000502), miR-200b (Assay ID: 001800), miR-221 (Assay ID: 000524) and miR-222 (Assay ID: 002276).

### Primer Validation

Primer pairs were designed according to standard qPCR criteria, including similar annealing temperatures between forward and reverse primers, GC content close to 50%, low self-complementarity, and minimal primer-dimer formation. Whenever possible, primers were designed to span different exons. Primer specificity was verified by melting curve analysis and agarose gel electrophoresis, which consistently showed a single amplification product of the expected size. Amplification efficiency was determined for each qPCR assay and ranged between 1.8 and 2.0.

### Statistical Analysis

All data are reported as mean ± S.E.M. Normal distribution of data and homogeneity of variances across groups were assessed using the Shapiro–Wilk test and Levene's test, respectively. Differences between groups were assessed by one-way analysis of variance (ANOVA), followed by the least significant difference (LSD) post hoc test. When homogeneity of variances was violated, variables were logarithmically transformed. Single comparisons were evaluated using Student’s *t-*test. Bivariate Pearson correlations were calculated between miRNAs and mRNA levels of genes related to lipid metabolism. Statistical significance was set at *p* ≤ 0.05. All analyses were carried out using IBM SPSS Statistics for Windows, Version 21.0. (IMB Corp., New York, USA).

## Results

### Study 1

The panel of 18 bioactive compounds is shown in Table 1. Compounds showing an apparent increase in leptin mRNA expression in the initial screening were selected for further qPCR analysis. The effects of the selected treatments, including OA, alpha-linolenic acid (ALA), propionic acid (PA), N-acetyl-glucosamine (NAG), betaine (Bet), leucin (Leu), homocysteine (Hcy), and hyaluronic acid (HA), are shown in Fig. 1. OA treatment at 200 µM significantly increased leptin mRNA levels compared to controls (Student’s t-test), as did Bet at 20 µM (one-way ANOVA). Treatment with 625 μM Leu showed a non-significant trend toward higher leptin expression (p < 0.10, one-way ANOVA). All other treatments did not affect leptin mRNA levels.

The general characterization of markers of MG differentiation, as well as adiponectin, is shown in Supplementary Table S2. Regarding adiponectin gene expression, none of the treatments assayed increased *Adipoq* mRNA levels. Treatments with 100 µM Bet or 10 µM Hcy significantly decreased *Adipoq* mRNA levels compared with controls. *Csn2* expression was analyzed as a validated biomarker for correct endocrine function in MEO [21]. *Csn2* mRNA levels increased after treatment with Bet (100 µM), NAG (7.5 mM), Hcy (50 µM), and Leu (625 µM) (Student’s t-test or one-way ANOVA). Fatty acids treatments modulated the expression of other casein-related genes. Specifically, higher mRNA levels relative to controls were found for *Csn1* (with PA treatment), *Csn1a2* (with ALA, PA, and OA treatments), and *Csn3* (with PA and OA treatments) (Student’s t-test). Moreover, amino acid-related compounds upregulated *Lalba* and *Prlr* mRNA expression. Specifically, Hcy (50 µM) and Leu (625 µM) increased *Lalba* mRNA levels compared with controls (one-way ANOVA). Likewise, Bet (at both doses), Hcy (50 µM), and Leu (625 µM) led to higher *Prlr* mRNA levels compared with control wells (one-way ANOVA). In addition, treatment with hyaluronic acid (10 mg/L) and NAG (7.5 mM) also increased *Prlr* gene expression (Student’s t-test). Finally, *Cns3* mRNA levels decreased after treatment with Hcy at 10 µM but increased after treatment with Hcy at 50 µM (one-way ANOVA).

### Study 2

**Fig. 2:**
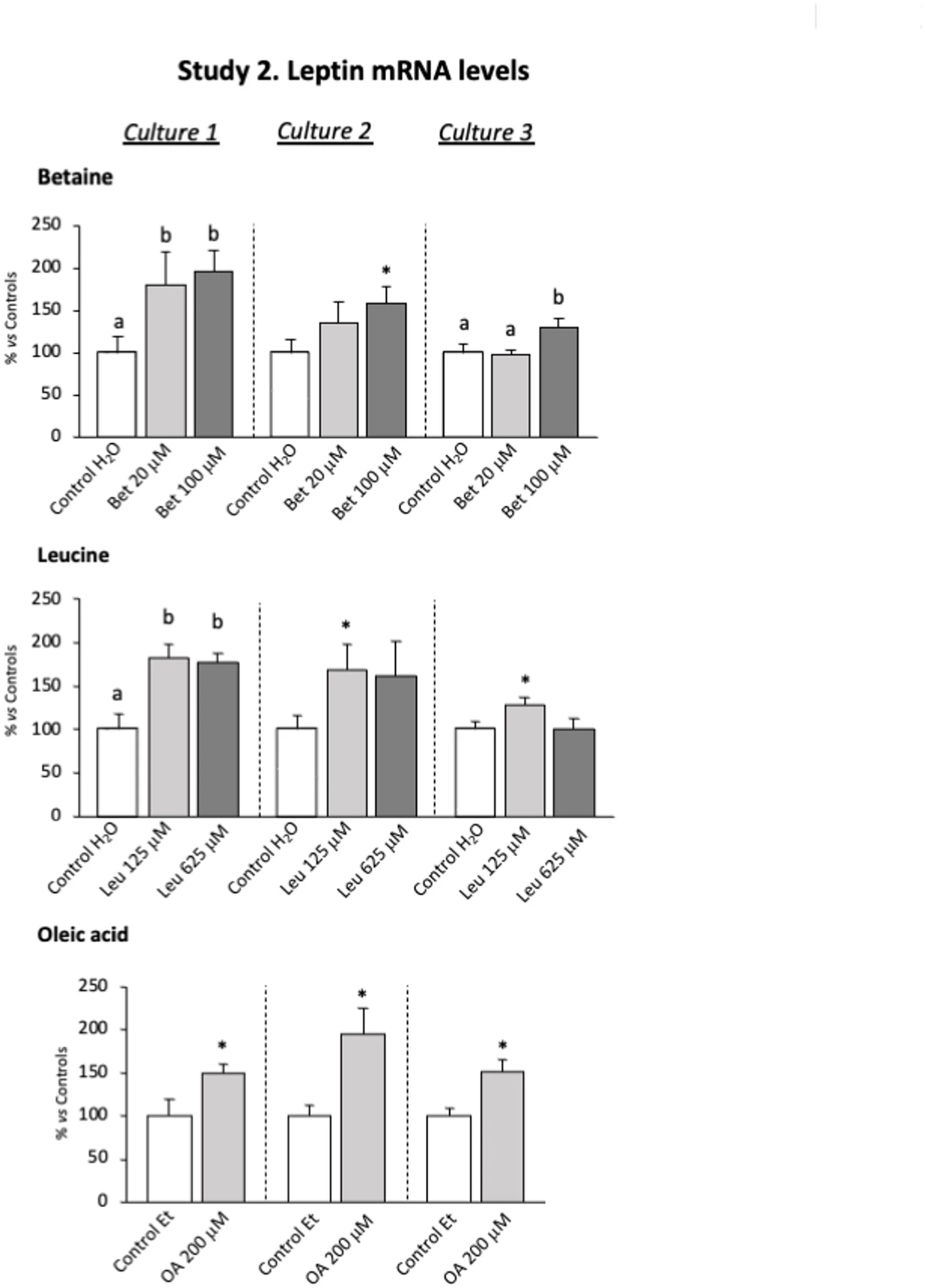
Study 2. mRNA Expression of Leptin in Rat MEOs Treated with Three Selected Bioactive Compounds at Different Doses Across Three Independent Cultures. Data are Mean ± S.E.M. (*n* = 7–15) and are Expressed Relative to the Corresponding Control for Each Compound. Statistics: Student’s t-test Significant Differences: * vs Control. Threshold Significance was Set at *p* ≤ 0.05. Acronyms: Bet, Betaine; Leu, Leucine; Et, Ethanol; and OA, Oleic Acid

The results of leptin mRNA levels following treatment in the three independent cultures are shown in Fig. 2. Notably, treatment with OA (200 µM), Bet (100 µM), and Leu (125 µM) significantly increased leptin gene expression compared with controls in all three cultures (Student’s t-test or one-way ANOVA). Bet at 20 µM and Leu at the highest dose (625 µM) significantly increased leptin mRNA levels only in the first culture (Student’s t-test or one-way ANOVA).

### Study 3

**Fig. 3:**
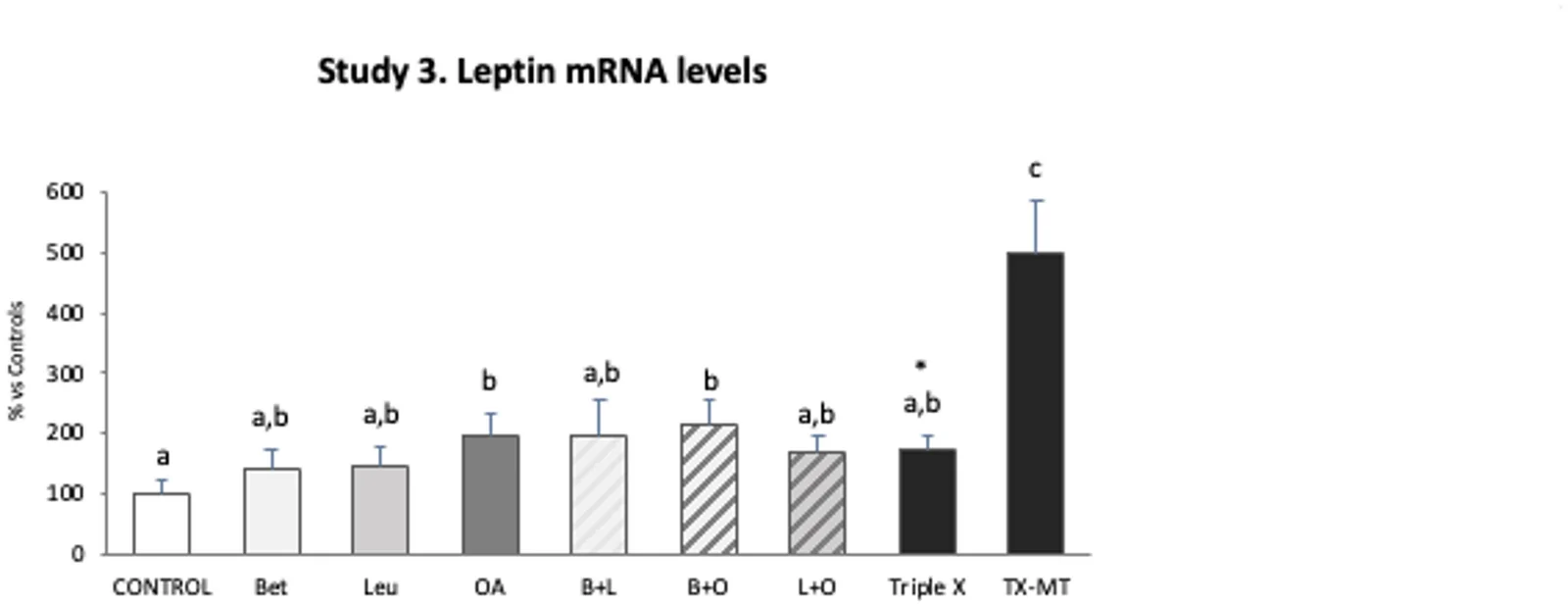
Study 3. mRNA eXpressions of Leptin in Rat MEOs Treated with Three Selected Bioactive Compounds, Alone or in Combination. Data are Mean ± S.E.M. (*n* = 4–10) and are Expressed Relative to the Corresponding Control for Each Compound. Statistics: Student’s t-test Significant Differences: * vs Control. Threshold Significance was Set at *p* ≤ 0.05. Acronyms: BEt, Betaine; Leu, Leucine; OA, Oleic Acid; B + L, Bet + Leu; B + O, Bet + OA; L + O; Leu + OA; Triple X, Bet + Leu + OA; and TX-MT, 7-Day Treatment with Bet + Leu + OA

Leptin mRNA levels following acute (24 h) treatment and mid-term treatment (7 days; Triple X combination only: Bet, Leu, and OA) are shown in Fig. 3. Treatment with OA alone or in combination significantly increased leptin gene expression compared with controls (Student’s t-test). Neither the combined treatment with Bet and Leu nor the individual treatments caused a significant increase in leptin mRNA levels. Mid-term treatment with the Triple X combination resulted in the highest leptin expression levels (fold change increases of 2.5 and 2.9 *vs.* OA and Triple X acute treatments, respectively; p < 0.05, Student’s t-test).

### Study 4

#### Leptin, Adiponectin, and Beta-casein Gene Expression

**Fig. 4:**
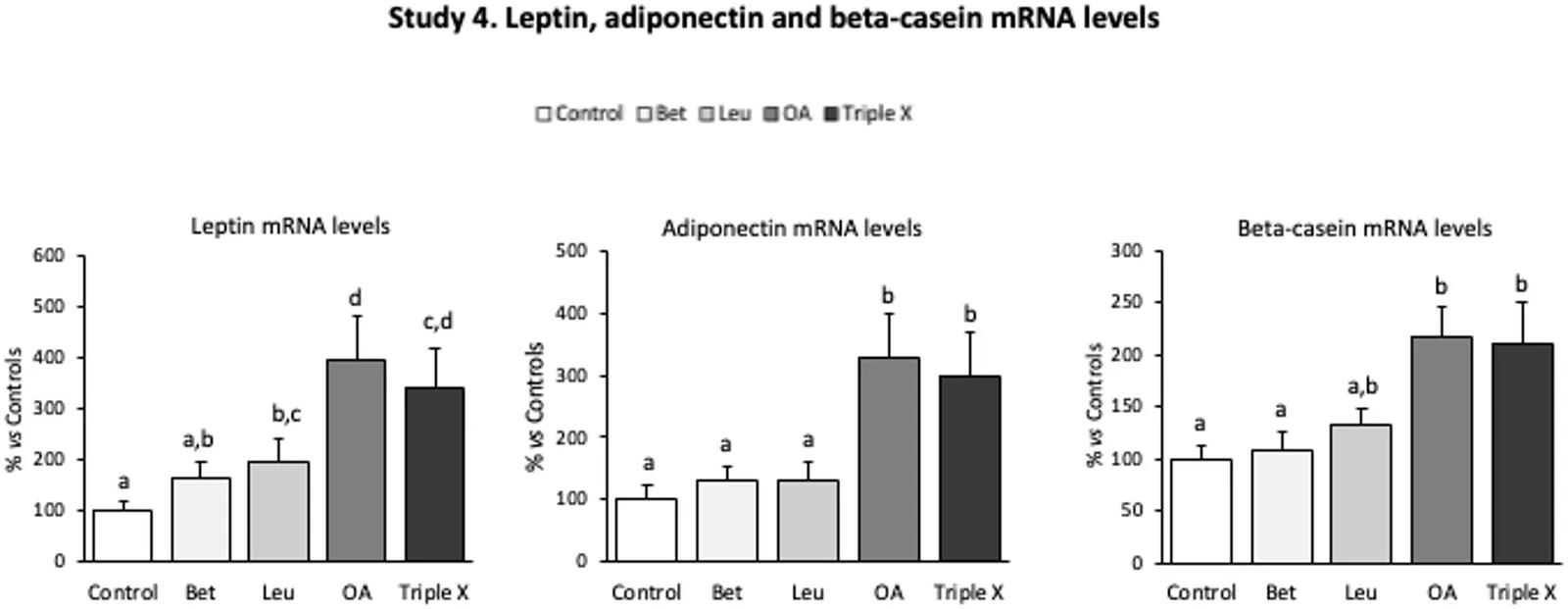
Study 4. mRNA Expressions of (**A**) Leptin, **B** Adiponectin and (**c**) Beta-casein (*Csn2*) in Rat MEOs Treated with Three Selected Bioactive Compounds, Alone or in Combination. Data are Mean ± S.E.M. (*n* = 8–10) and are Expressed Relative to the Corresponding Control for Each Compound. The Double Combination was Formed by OA and Bet, While the Triple Combination Administered the Three of Them. Statistics: LSD Post Hoc One-way ANOVA test: Data Not Sharing a Common Letter are Significantly Different. Threshold Significance was Set at *p* ≤ 0.05. Acronyms: Bet, Betaine; Leu, Leucine; OA, Oleic Acid; and Triple X, Bet + Leu + OA

Leptin, adiponectin, and beta-casein (*Csn2*) mRNA expression levels after a 7-day mid-term treatment with the three individual compounds from study 3 and the Triple X combination are presented in Fig. 4. Mid-term treatment with OA (200 µM) alone or with the triple combination significantly increased leptin, adiponectin, and *Csn2* mRNA levels (one-way ANOVA). Leu (125 µM) significantly increased leptin mRNA levels compared with controls; however, this effect was less pronounced than that observed with OA alone or in combination (one-way ANOVA). No significant changes were found in adiponectin or *Csn2* mRNA levels following individual treatment with Bet or Leu, nor in leptin mRNA levels after individual Bet treatment.

#### miRNA Expression and Target Genes

**Fig. 5:**
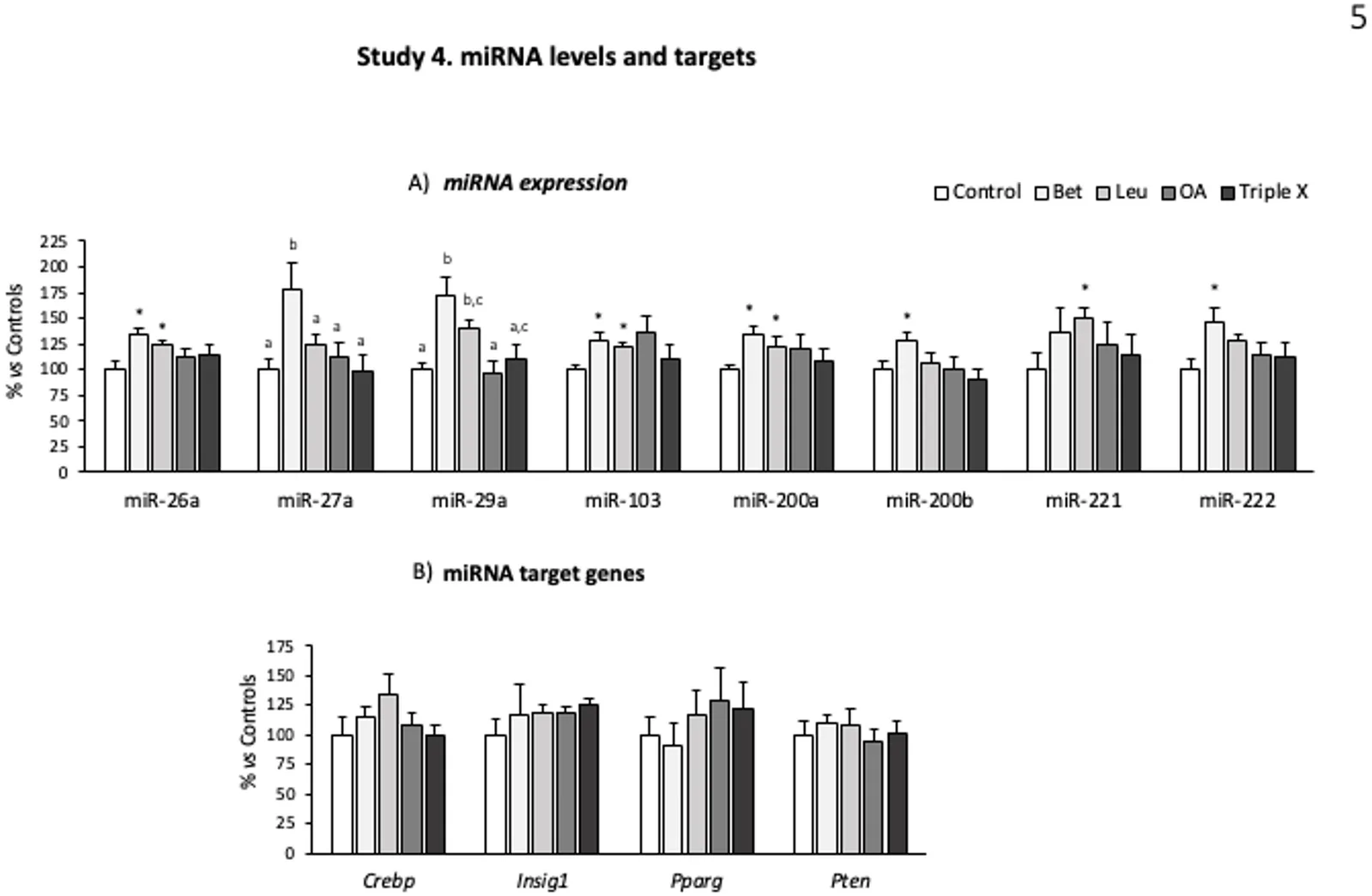
Study 4. Expression of Several miRNAs Involved in Leptin Signaling in Rat MEOs Treated with Three Selected Bioactive Compounds, Alone or in Combination. Data are Mean ± S.E.M. (*n* = 7–10) and are Expressed Relative to the Corresponding Control for Each Compound. The Double Combination Was Formed by OA and Bet, while the Triple Combination Mixed the Three of Them. Statistics: LSD Post Hoc One-way ANOVA Test: Data Not Sharing a Common Letter Are Significantly Different; Student’s t-test Significant Differences: * vs Control; ∩ vs Bet. Threshold Significance was Set at *p* ≤ 0.05. Acronyms and Abbreviations: Bet, Betaine; Leu, Leucine; OA, Oleic Acid; Triple X, Bet + Leu + OA; cAMP-binding Protein (*Crebp*); insulin-induced gene 1 (*Insig1*); Peroxisome Proliferator-activated Receptor Gamma (*Pparg*); and Phosphatase and Tensin Homolog (*Pten*)

The expression levels of the selected miRNAs are displayed in Fig. 5A. Statistical analysis indicated that OA, either alone or in combination, did not significantly affect miRNA expression compared with controls. In contrast, treatment with Bet or Leu significantly increased the expression of most of the miRNAs in comparison to controls. Specifically, Bet-treated cells presented significantly higher levels of miR-26a, miR-27a, miR-29a, miR-103, miR-200a, miR-200b, and miR-222 compared with controls (one-way ANOVA or Student’s t-test). Similarly, Leu treatment significantly increased the expression of miR-26a, miR-29a, miR-103, miR-200a, and miR-221 compared to controls (Student’s t-test). Notably, co-treatment of Bet or Leu with OA significantly attenuated the increases observed with the individual treatments.

The expression levels of selected target genes of the analyzed miRNAs are shown in Fig. 5B. No significant changes were detected in the expression levels of these target genes under any treatment condition.

#### mRNA Profile of Lipid and One-carbon Metabolism-related Genes

**Fig. 6:**
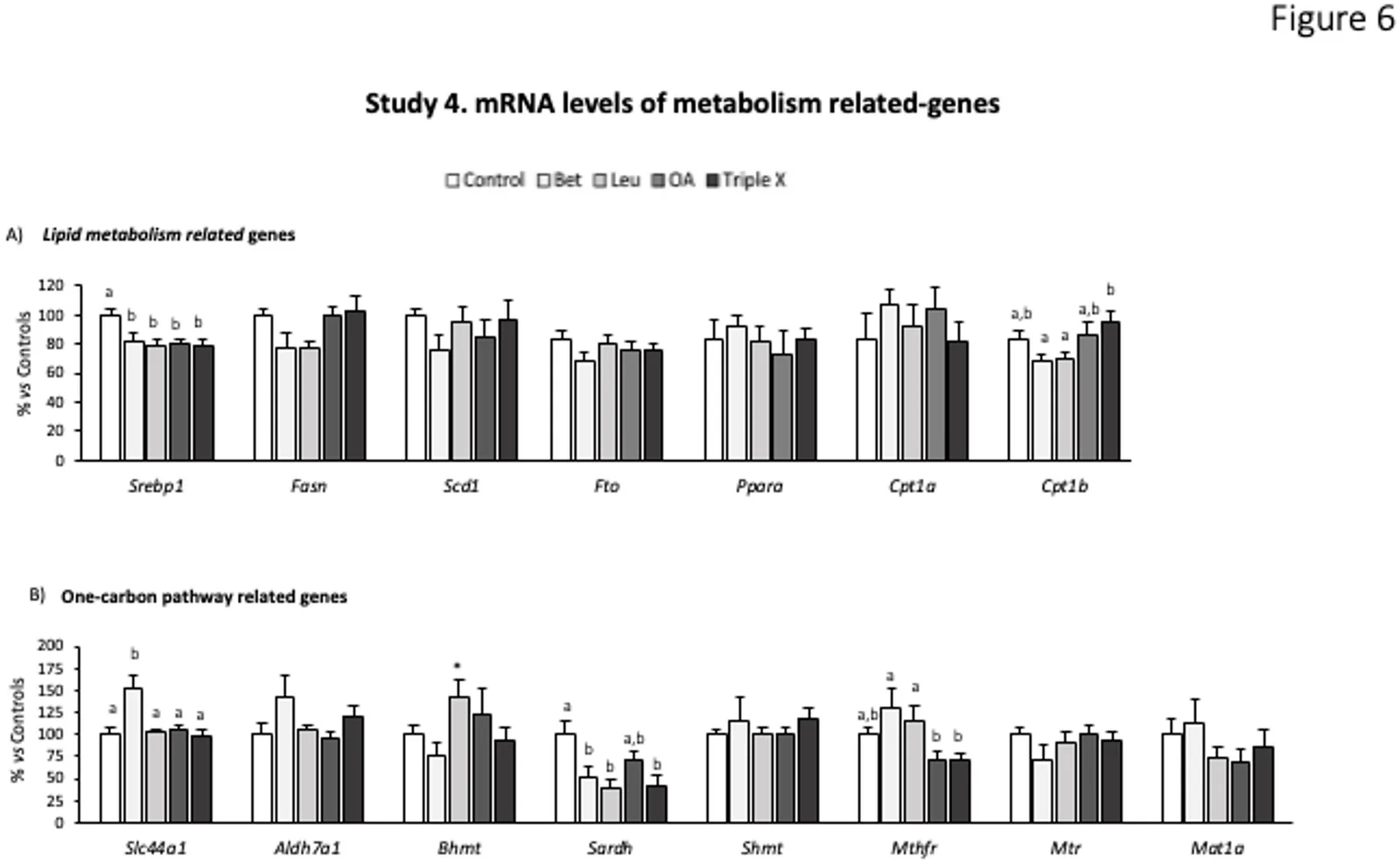
Study 4. mRNA Expression of (**A**) Selected Target genes for miRNAs analyzed, **B** Genes Related to Lipid Metabolism and (**C**) Genes Involved in One-carbon Metabolism Processes in Rat MEOs Treated with Three Selected Bioactive Compounds, Alone or in Combination. Data are Mean ± S.E.M. (*n* = 7–10) and are Expressed Relative to the Corresponding Control for Each Compound. Statistics: LSD Post Hoc One-way ANOVA test: Data Not Sharing a Common Letter Are Significantly Different; Student’s t-test Significant Differences: * vs Control; # vs Oleic acid; +, vs Leucine. Threshold Significance was Set at *p* ≤ 0.05. Acronyms and Abbreviations: Bet, Betaine; Leu, Leucine; OA, Oleic acid; Triple X, Bet + Leu + OA; Fat Mass and Obesity-associated Protein (*Fto*); Peroxisome Proliferator-activated Receptor Alpha (*Ppara*); Carnitine Palmitoyltransferase 1 A (*Cpt1a*) and 1B (*Cpt1b*); peroxisome Proliferator-activated Receptor γ co-activator 1 alpha (*Pgc1a*); sterol Regulatory Element Binding Protein 1 (*Srebp1*); Fatty Acid Synthase (*Fasn*); stearoyl-CoA desaturase (*Scd1*); Solute Carrier Family 44 Member 1 (*Slc44a1*); Aldehyde Dehydrogenase 7 Family Member A1 (*Aldh7a1*); Betaine-homocysteine S-Methyltransferase (*Bhmt*); Sarcosine Dehydrogenase (*Sardh*); Serine Hydroxymethyltransferase (*Shmt*); Methylenetetrahydrofolate Reductase (Mthfr); Methyltetrahydrofolate-homocysteine Methyltransferase (*Mtr*); and mEthionine Adenosyl-transferase 1a (*Mat1a)*

The mRNA levels of selected genes related to lipid metabolism and one-carbon (1C) metabolism are presented in Fig. 6A-B.

Regarding lipid metabolism (Fig. 6A), *Srebp1* mRNA levels were significantly reduced by all the treatments compared with controls (Student’s t-test), suggesting a decreased lipogenic capacity. In addition, the triple combination significantly increased *Cpt1b* mRNA levels compared with Bet and Leu administered individually (one-way ANOVA). No significant differences were observed in the expression of the remaining lipid-related genes among treatments or relative to controls.

Given that the 1 C pathway is influenced by lipid metabolism [22] and mediates numerous biological processes essential for the proper neonatal development [23], we evaluated the expression of 1C-related genes to further comprehend the potential effects of leptin and/or these compounds (Fig. 6B).

*Mthfr* mRNA levels were significantly decreased after OA treatment alone or in combination, compared with controls and with Bet or Leu administered individually (one-way ANOVA). *Slc44a1* expression was significantly higher following Bet treatment in comparison to the other groups, including the triple combination (one-way ANOVA). *Sardh* mRNA levels were significantly reduced by Bet, Leu, and triple combination compared with controls (one-way ANOVA), whereas OA treatment induced a non-significant (28.8%) reduction relative to controls. Finally, Leu treatment significantly increased *Bhmt* expression compared with controls (Student’s t-test); this effect was abolished in the triple combination.

#### Bivariate Pearson Correlations Between miRNAs and Lipid Metabolism-related Gene Expression

Supplementary Table S3 summarizes the significant correlations found between miRNA expression and the mRNA levels of lipid metabolism-related genes. Notably, *Scd1*, *Fasn*, *Srebp1*, *Fto,* and *Cpt1b* mRNA levels were negatively correlated with most of the analyzed miRNAs, whereas *Cpt1a* expression showed significant positive correlations.

## Discussion

The composition of breast milk is a key determinant of perinatal programming and future metabolic health. Beyond macronutrients, variations in micronutrients and bioactive components have been associated with long-term effects on infant adiposity and body weight regulation [24]. Among these, milk leptin and adiponectin have attracted particular attention due to their potential roles in metabolic programming during critical stages of development [15, 24]. The levels of these and other milk-derived components depend, at least in part, on mammary gland-specific production during lactation, which is strongly influenced by maternal nutritional status [24].

In this context, this study identified dietary bioactive compounds capable of modulating leptin expression in MEO and characterized associated molecular pathways. The results show that selected nutrients, particularly OA, Leu, and Bet, differentially regulate leptin gene expression and metabolic pathways in primary mammary epithelial cells. These findings support the use of MEO as an in vitro model for studying nutrient regulation of mammary gland differentiation and function [18].

In brief, OA, Bet, and Leu treatments showed distinct effects on gene expression in MEO. OA, alone or in combination, consistently increased leptin gene expression, whereas Bet and Leu induced an acute increase in leptin expression that was not maintained after mid-term treatment, despite their effects on the miRNA profile.

Among the compounds tested, OA produced the most consistent increase in leptin gene expression across experimental conditions. Both acute and mid-term OA treatments increased leptin mRNA levels, either alone or in combination, supporting a direct role for this monounsaturated fatty acid in the regulation of leptin expression in mammary epithelial cells. Similar effects of OA on leptin expression have been reported in other cell types [25–28]. For example, Malodobra-Mazur et al. [28] found that OA treatment of 3T3-L1 adipocytes at comparable concentrations (250 and 500 µM) increased both leptin mRNA expression and leptin secretion. In the same study, the highest OA dose reduced adiponectin secretion without affecting its mRNA levels, in contrast to the results observed here.

One mechanism proposed to contribute to OA-mediated regulation of leptin expression involves changes in DNA methylation [28, 29]. Reduced global DNA methylation has been reported in 3T3-L1 adipocytes treated with 500 µM OA [28], as well as in human monocytes exposed to 100 µM OA for 24 h [30]. Although DNA methylation was not assessed in the present study, OA treatment, alone or in combination, reduced the expression of *Mthfr,* which encodes a key enzyme in the generation of S-adenosylmethionine (SAM), the universal methyl donor for all intracellular methylation reactions [29]. This finding may suggest a reduced capacity for homocysteine remethylation to methionine under OA treatment, potentially influencing methylation-dependent gene regulation.

OA also increased adiponectin and *Csn2* expression during mid-term treatment, suggesting that its effects were not limited to leptin regulation and may reflect an overall enhancement of mammary epithelial endocrine function and differentiation status [20]. In addition, since beta-casein is one of the most abundant proteins in milk [31], these findings, together with the increased levels of other caseins under acute conditions, suggest that OA supplementation may influence milk protein composition, warranting further investigation into its potential impact on milk quality. Reduced *Srebp1* expression following OA treatment suggest reduced lipogenic activity and a shift toward a more oxidative metabolic profile. This is consistent with the increase in *Cpt1b* mRNA levels observed with the triple combination treatment, suggesting that OA activity may be potentiated when administered as part of the mixture, although the underlying mechanisms remain unclear. These findings partially agree with previous in vivo observations [15]. Dams receiving a diet enriched with OA, Bet, and Leu during lactation exhibited higher leptin levels in milk, increased leptin expression in WAT, and reduced lipogenic capacity [15]. However, adiponectin expression in the mammary gland was reduced in vivo [15], contrasting with the upregulation observed in our in vitro MEO model. This difference may reflect the greater complexity of in vivo regulation, including systemic ant tissue-specific interactions that are not fully captured in isolated cell cultures.

Leu and Bet showed more variable effects on leptin expression than OA. Both compounds increased leptin mRNA levels under specific conditions, but their effects depended on dose, culture conditions, and treatment duration. Leu, a known activator of mTORC1 signaling [32], may influence leptin expression indirectly through nutrient-sensing pathways associated with cellular growth and differentiation. The modest induction of leptin observed with Leu alone, together with its ability to enhance *Csn2* and lactalbumin expression (under acute conditions), supports its potential role in promoting mammary gland maturation [20] rather than acting as a primary regulator of leptin expression. Bet, a methyl donor involved in one-carbon metabolism [29], also showed dose-dependent effects. While Bet treatment increased leptin expression under some acute conditions, higher doses reduced adiponectin expression. Moreover, the observed modulation of one-carbon metabolism–related genes by individual Bet and Leu treatments, including changes in *Slc44a1*, *Sardh*, and *Bhmt* mRNA levels, is consistent with an effect of these nutrients on one-carbon metabolism-related pathways, although the underlying mechanisms were not investigated in the present study. Overall, the relatively modest changes observed for Bet and Leu suggest that additional regulatory mechanisms may contribute to their biological effects.

The analysis of miRNA expression revealed additional treatment-dependent effects. OA did not significantly alter the expression of the selected miRNAs, whereas Bet and Leu increased the levels of several miRNAs associated with metabolic regulation. The reversal of miRNA induction by OA in the Triple X treatment suggests that OA may exert a dominant regulatory effect, potentially mitigating miRNA-mediated repression of metabolic genes. The analyzed miRNAs were selected based on their potential involvement in the modulation of leptin and/or adiponectin or being modulated by them. Indeed, miR-26a, miR-27a, miR-103, miR-200a, and miR-222 levels have been associated with leptin and adiponectin levels in milk in human and animal studies [15–17], and miR-200a/−200b have been reported as leptin targets in yellow catfish [33]. mir-221 has also been described as leptin-responsive in human preadipocytes [34]. Correlation analysis showed a negative association between selected miRNAs and the expression of lipid metabolism-related genes, which ultimately regulate adiposity and are therefore related to leptin and adiponectin synthesis. For example, mir-29a regulates SCD [35, 36], and mir-221 has been described as a regulator of lipid metabolism in mammary epithelial cells, targeting genes related to lipid synthesis, such as *Fasn* [36]. In the present study, mir-221 expression was negatively correlated with the expression of lipogenic genes (*Srebp1*, *Scd1*, and *Fasn*), suggesting a potential role for miR-221 in modulating MG lipid metabolism, although functional validation is required.

While the present study does not establish a causal relationship between adipokine expression and miRNA modulation, distinct patterns were observed. Treatments that increased leptin and adiponectin expression (OA alone or in combination) did not affect the expression of the analyzed miRNAs, whereas treatments that did not increase adipokine expression (Bet and Leu alone) were associated with changes in miRNA levels. In vivo studies have reported downregulation of the same miRNAs in the milk of lactating dams fed a diet enriched with OA, Bet, and Leu [16]. However, the mechanisms underlying these differences remain unclear. Supporting this idea, the literature indicates that the absence of leptin upregulates miR-29a, miR­200a, and miR-222 in the hypothalamus of newborn rats [37], whereas it slightly downregulates miR­26a in two obese mouse models [38]. Moreover, miR-27a is known to inhibit lipogenesis [39], and leptin has been shown to upregulate miR-27a in hepatic cells [40]. Overall, these findings suggest a potential association between leptin expression and miRNA levels, but further studies are needed to confirm these hypotheses. It cannot be excluded that the lack of miRNA induction in the triple combination reflects interactive effects between Leu and Bet.

Altogether, the present study demonstrates that selected bioactive compounds (OA, Bet, and Leu), either alone or in combination, modulate the gene expression profile of rat MEO relevant to mammary gland function. OA, alone or in combination, consistently increased leptin, adiponectin, and *Csn2* mRNA expression, while partially reducing the expression of genes related to lipogenesis and one-carbon metabolism. In contrast, Bet and Leu primarily affected miRNA expression and selected one-carbon metabolism–related genes, with limited effects on adipokine or lipid metabolism–related mRNA levels. Overall, these findings support the use of a controlled 3D in vitro mammary epithelial model as a proof-of-concept platform to investigate nutrient–gene interactions relevant to lactation before in vivo validation. Future studies should address the translational relevance of these findings.

## Supplementary Information

Supplementary Material 1.

## Data Availability

No datasets were generated or analysed during the current study.
