# Supplementary material for: Reprogramming of Metabolic and Adipokine-related Gene Expression in 3D Mammary Epithelial Organoids by Dietary Bioactive Compounds
Source: J Mammary Gland Biol Neoplasia. 2026 Jul 29;31(1):24. doi: 10.1007/s10911-026-09612-z (PMC13696566; doi:10.1007/s10911-026-09612-z)
Supplement: Supplementary file 1 [file 10911_2026_9612_MOESM1_ESM.pdf]

Supplementary material.

Reprogramming of metabolic and adipokine-related gene expression in 3D mammary epithelial organoids by dietary bioactive compounds

Authors: Mariona Palou[1,2,3,4,5], Marta Alonso-Bernáldez[1,5], Andreu Palou-March[1,2,3,4,5], Andreu Palou[1,2,3,4,5], Catalina Picó[1,2,3,4,5*], and Francisca Serra[1,2,3,4,5].

[1] Laboratory of Molecular Biology, Nutrition and Biotechnology (Group of Nutrigenomics, Biomarkers and risk Evaluation), University of the Balearic Islands, Palma, Spain.

[2] Institut d'Investigació Sanitària Illes Balears (IdISBa), Palma, Spain.

[3] CIBER de Fisiopatología de la Obesidad y Nutrición (CIBEROBN), Instituto de Salud Carlos III, Madrid, Spain

[4] Artificial Intelligence Research Institute of the Balearic Islands (IAIB), Palma, Spain.

[5] Alimentómica S.L. (Spin off no. 001 from UIB), Parc Bit, 07120 Palma, Spain.

* Correspondence: cati.pico@uib.es

Supplementary Table S1. Rationale for the selection and doses of candidate nutritional compounds

Compound	Category	Main dietary sources / presence in milk	Reported biological relevance	References
		**Fatty acids**		
Oleic acid	Monounsaturated fatty acid (ω-9)	Olive oil, rapeseed oil, other vegetable oils	Cardiovascular and hepatic health; maintenance of normal LDL-cholesterol levels	[1-8]
Linoleic acid (LA)	Polyunsaturated fatty acid (ω-6, essential)	Sunflower, safflower, corn, soy oils; nuts; cereals	Regulation of cholesterol and triglycerides; immune function	[1-3,6,7,9-11]
α-Linolenic acid (ALA)	Polyunsaturated fatty acid (ω-3, essential)	Flaxseed, linseed oil, leafy greens, Brussels sprouts	Cardiovascular protection; neurodevelopment during pregnancy and lactation	[1,2,10-13]
Conjugated linoleic acid (CLA)	Modified fatty acid	Ruminant-derived foods; supplements	Appetite regulation; reduced body weight gain	[14-21]
Eicosapentaenoic acid (EPA)	Long-chain PUFA (ω-3)	Fatty fish; crustaceans; present in human milk	Regulation of lipemia; cardiovascular function; precursor of eicosanoids	[1,2,4,11,22,23]
Docosahexaenoic acid (DHA)	Long-chain PUFA (ω-3)	Fatty fish; present in human milk	Brain and visual development; anti-inflammatory effects	[1,12,22-25]
Propionic acid	Short-chain fatty acid	Produced by colonic fermentation of dietary fiber	Modulation of leptin expression; metabolic regulation	[26]
Butiric acid	Short-chain fatty acid	Produced by colonic fermentation of dietary fiber	Metabolic regulation	[26,27]
		**Essential and non-essential amino acids**		
L-Leucine	Essential amino acid (BCAA)	Meat, fish, soy, legumes, cereals, nuts	Muscle protein synthesis; glycemic control	[28-35]
L-Glutamine	Non-essential amino acid	Protein-rich animal and plant foods	Maintenance of muscle mass; cellular stress response	[28,29,36-38]
L-Methionine	Essential sulfur-containing amino acid	Fish, meat, sesame seeds, nuts	Methyl-group metabolism; cardiovascular health	[19,28,29,39-41]
Homocysteine	Sulfur-containing amino acid (non-proteinogenic)	Intermediate of methionine metabolism	Cardiovascular and cerebrovascular risk marker	[41-47]

Compound	Category	Main dietary sources / presence in milk	Reported biological relevance	References
**Methionine cycle intermediates and methyl donors**				
Choline	Methyl donor; B-vitamin–like nutrient	Liver, eggs, fish, soy, cruciferous vegetables	Membrane integrity; neurotransmitter synthesis; methyl metabolism	45,48-51
Betaine	Methyl donor	Fish, beetroot, legumes	Reduction of circulating homocysteine levels, adipokines modultation	45,48-50,52,53
Folic acid	Water-soluble B vitamin	Legumes, leafy greens, fortified cereals, nuts	DNA synthesis; cell division; growth and development	45,48-50,54,55
**Amino sugars and glycosaminoglycans**				
D-Glucosamine	Amino sugar	Shellfish exoskeletons, fungi	Protein and lipid glycosylation; joint health	56-58
N-acetyl-D-glucosamine	Amino sugar derivative	Structural polysaccharides	Immune modulation; precursor of hyaluronic acid	59-61
Hyaluronic acid	Glycosaminoglycan	Synovial fluid, connective tissue	Structural function; joint and tissue health	62-64

List of References of S1:

1. Priego T, Sanchez J, Garcia AP, Palou A, Pico C. Maternal dietary fat affects milk fatty acid profile and impacts on weight gain and thermogenic capacity of suckling rats. *Lipids*. May 2013;48(5):481-95. doi:10.1007/s11745-013-3764-8
2. Laraichi S, Parra P, Zamanillo R, El Amarti A, Palou A, Serra F. Dietary supplementation of calcium may counteract obesity in mice mediated by changes in plasma fatty acids. *Lipids*. Aug 2013;48(8):817-26. doi:10.1007/s11745-013-3798-y
3. Zaman MM, Martin CR, Andersson C, et al. Linoleic acid supplementation results in increased arachidonic acid and eicosanoid production in CF airway cells and in cftr-/- transgenic mice. *Am J Physiol Lung Cell Mol Physiol*. Nov 2010;299(5):L599-606. doi:10.1152/ajplung.00346.2009]
4. Solberg R, Aas V, Thoresen GH, et al. Leptin expression in human primary skeletal muscle cells is reduced during differentiation. *J Cell Biochem*. Sep 1 2005;96(1):89-96. doi:10.1002/jcb.20521
5. Granados N, Amengual J, Ribot J, Palou A, Bonet ML. Distinct effects of oleic acid and its trans-isomer elaidic acid on the expression of myokines and adipokines in cell models. *Br J Nutr*. Apr 2011;105(8):1226-34. doi:10.1017/S0007114510004885
6. Pauloin A, Chat S, Pechoux C, et al. Oleate and linoleate stimulate degradation of beta-casein in prolactin-treated HC11 mouse mammary epithelial cells. *Cell Tissue Res*. Apr 2010;340(1):91-102. doi:10.1007/s00441-009-0926-3
7. Sigurdson SL, Ip MM. Casein accumulation by rat mammary epithelial cells grown within a reconstituted basement membrane is modulated by fatty acids in a hormone- and time-dependent manner. *Exp Cell Res*. Oct 1993;208(2):333-43. doi:S0014-4827(83)71254-1
8. EFSA. European Food Safety Authority. Panel on Dietetic Products, Nutrition and Allergies (NDA). Scientific Opinion on the substantiation of health claims related to the replacement of mixtures of saturated fatty acids (SFAs) as present in foods or diets with mixtures of monounsaturated fatty acids (MUFAs) and/or mixtures of polyunsaturated fatty acids (PUFAs), and maintenance of normal blood LDL-cholesterol concentrations (ID 621, 1190, 1203, 2906, 2910, 3065) pursuant to Article 13(1) of Regulation (EC) No 1924/2006. *The EFSA Journal*. 2011;9:pp: 2.069.
9. Harant-Farrugia I, Garcia J, Iglesias-Osma MC, Garcia-Barrado MJ, Carpene C. Is there an optimal dose for dietary linoleic acid? Lessons from essential fatty acid deficiency supplementation and adipocyte functions in rats. *J Physiol Biochem*. Feb 1 2014;doi:10.1007/s13105-014-0315-6

10. Cammisotto PG, Gelinas Y, Deshaies Y, Bukowiecki LJ. Regulation of leptin secretion from white adipocytes by free fatty acids. *Am J Physiol Endocrinol Metab*. Sep 2003;285(3):E521-6. doi:10.1152/ajpendo.00052.2003
11. EFSA. European Food Safety Authority. EFSA Panel on Dietetic Products, Nutrition, and Allergies (NDA). Scientific Opinion on the substantiation of health claims related to EPA, DHA, DPA and maintenance of normal blood pressure (ID 502), maintenance of normal HDL-cholesterol concentrations (ID 515), maintenance of normal (fasting) blood concentrations of triglycerides (ID 517), maintenance of normal LDL-cholesterol concentrations (ID 528, 698) and maintenance of joints (ID 503, 505, 507, 511, 518, 524, 526, 535, 537) pursuant to Article 13(1) of Regulation (EC) No 1924/2006. *The EFSA Journal*. 2009;7(9):1.263, pp:26.
12. Thies F, Nebe-von-Caron G, Powell JR, Yaqoob P, Newsholme EA, Calder PC. Dietary supplementation with eicosapentaenoic acid, but not with other long-chain n-3 or n-6 polyunsaturated fatty acids, decreases natural killer cell activity in healthy subjects aged >55 y. *Am J Clin Nutr*. Mar 2001;73(3):539-48.
13. EFSA. European Food Safety Authority. EFSA Panel on Dietetic Products, Nutrition and Allergies (NDA). Scientific Opinion on the substantiation of a health claim related to alpha-linolenic acid and contribution to brain and nerve tissue development pursuant to Article 14 of Regulation (EC) No 1924/2006. *The EFSA Journal*. 2011;9(4):2.130, pp:8.
14. Mele MC, Cannelli G, Carta G, et al. Metabolism of c9,t11-conjugated linoleic acid (CLA) in humans. *Prostaglandins Leukot Essent Fatty Acids*. Aug 2013;89(2-3):115-9. doi:10.1016/j.plefa.2013.05.005
15. Abdelmagid SA, Clarke SE, Wong J, et al. Plasma concentration of cis9trans11 CLA in males and females is influenced by SCD1 genetic variations and hormonal contraceptives: a cross-sectional study. *Nutr Metab (Lond)*. Jul 18 2013;10(1):50. doi:1743-7075-10-50
16. Gradzka I, Sochanowicz B, Brzoska K, et al. Cis-9,trans-11-conjugated linoleic acid affects lipid raft composition and sensitizes human colorectal adenocarcinoma HT-29 cells to X-radiation. *Biochim Biophys Acta*. Jan 2013;1830(1):2233-42. doi:10.1016/j.bbagen.2012.10.015
17. Liu JR, Li BX, Chen BQ, et al. Effect of cis-9, trans-11-conjugated linoleic acid on cell cycle of gastric adenocarcinoma cell line (SGC-7901). *World J Gastroenterol*. Apr 2002;8(2):224-9.
18. Selga E, Perez-Cano FJ, Franch A, et al. Gene expression profiles in rat mesenteric lymph nodes upon supplementation with conjugated linoleic acid during gestation and suckling. *BMC Genomics*. 2011;12:182. doi:10.1186/1471-2164-12-182
19. Riserus U, Vessby B, Arnlov J, Basu S. Effects of cis-9,trans-11 conjugated linoleic acid supplementation on insulin sensitivity, lipid peroxidation, and proinflammatory markers in obese men. *Am J Clin Nutr*. Aug 2004;80(2):279-83. doi:80/2/279
20. Parra P, Serra F, Palou A. Moderate doses of conjugated linoleic acid isomers mix contribute to lowering body fat content maintaining insulin sensitivity and a noninflammatory pattern in adipose tissue in mice. Research Support, Non-U.S. Gov't. *J Nutr Biochem*. Feb 2010;21(2):107-15. doi:10.1016/j.jnutbio.2008.10.010
21. Parra P, Palou A, Serra F. Moderate doses of conjugated linoleic acid reduce fat gain, maintain insulin sensitivity without impairing inflammatory adipose tissue status in mice fed a high-fat diet. *Nutr Metab (Lond)*. 2010;7:5. doi:10.1186/1743-7075-7-5
22. Perez-Matute P, Marti A, Martinez JA, et al. Eicosapentaenoic fatty acid increases leptin secretion from primary cultured rat adipocytes: role of glucose metabolism. *Am J Physiol Regul Integr Comp Physiol*. Jun 2005;288(6):R1682-8. doi:00727.2004
23. Hutchins-Wiese HL, Picho K, Watkins BA, et al. High-dose eicosapentaenoic acid and docosahexaenoic acid supplementation reduces bone resorption in postmenopausal breast cancer survivors on aromatase inhibitors: a pilot study. *Nutr Cancer*. 2014;66(1):68-76. doi:10.1080/01635581.2014.847964
24. EFSA. European Food Safety Authority. EFSA Panel on Dietetic Products, Nutrition, and Allergies (NDA). Scientific Opinion on the substantiation of health claims related to docosahexaenoic acid (DHA) and maintenance of normal (fasting) blood concentrations of triglycerides (ID 533, 691, 3150), protection of blood lipids from oxidative damage (ID 630), contribution to the maintenance or achievement of a normal body weight (ID 629), brain, eye and nerve development (ID 627, 689, 704, 742, 3148, 3151), maintenance of normal brain function (ID 565, 626, 631, 689, 690, 704, 742, 3148, 3151), maintenance of normal vision (ID 627, 632, 743, 3149) and maintenance of normal spermatozoa motility (ID 628) pursuant to Article 13(1) of Regulation (EC) No 1924/2006. *The EFSA Journal*. 2010;8(10):1734, pp:27.
25. Velten M, Britt RD, Jr., Heyob KM, Tipple TE, Rogers LK. Maternal Dietary Docosahexaenoic Acid Supplementation Attenuates Fetal Growth Restriction and Enhances Pulmonary Function in a Newborn Mouse Model of Perinatal Inflammation. *J Nutr*. Jan 22 2014;doi:jn.113.179259
6. Al-Lahham SH, Roelofsen H, Priebe M, et al. Regulation of adipokine production in human adipose tissue by propionic acid. Research Support, Non-U.S. Gov't. *Eur J Clin Invest*. May 2010;40(5):401-7. doi:10.1111/j.1365-2362.2010.02278.x
27. Li Z, Yi CX, Katiraei S, et al. Butyrate reduces appetite and activates brown adipose tissue via the gut-brain neural circuit. *Gut*. Jul 2018;67(7):1269-1279. doi:10.1136/gutjnl-2017-314050
28. Cynober LA. Plasma amino acid levels with a note on membrane transport: characteristics, regulation, and metabolic significance. *Nutrition*. Sep 2002;18(9):761-6. doi:S0899900702007803 [pii]
29. Muratsubaki H, Yamaki A. Profile of plasma amino Acid levels in rats exposed to acute hypoxic hypoxia. *Indian J Clin Biochem*. Oct 2011;26(4):416-9. doi:10.1007/s12291-011-0125-3
30. Lopez N, Sanchez J, Pico C, Palou A, Serra F. Dietary l-leucine supplementation of lactating rats results in a tendency to increase lean/fat ratio associated to lower orexigenic neuropeptide expression in hypothalamus. *Peptides*. Jul 2010;31(7):1361-7. doi:10.1016/j.peptides.2010.03.028]
31. Pereira MG, Baptista IL, Carlassara EO, Moriscot AS, Aoki MS, Miyabara EH. Leucine supplementation improves skeletal muscle regeneration after cryolesion in rats. *PLoS One*. 2014;9(1):e85283. doi:10.1371/journal.pone.0085283

32. Wing-Gaia SL, Gershenoff DC, Drummond MJ, Askew EW. Effect of leucine supplementation on fat free mass with prolonged hypoxic exposure during a 13-day trek to Everest Base Camp: a double-blind randomized study. *Appl Physiol Nutr Metab*. Mar 2014;39(3):318-323. doi:10.1139/apnm-2013-0319
33. Zampieri TT, Pedroso JA, Furigo IC, Tirapegui J, Donato J, Jr. Oral Leucine Supplementation Is Sensed by the Brain but neither Reduces Food Intake nor Induces an Anorectic Pattern of Gene Expression in the Hypothalamus. *PLoS One*. 2013;8(12):e84094. doi:10.1371/journal.pone.0084094
34. Garlick PJ. The role of leucine in the regulation of protein metabolism. Research Support, Non-U.S. Gov't Review. *J Nutr*. Jun 2005;135(6 Suppl):1553S-6S.
35. Layman DK. Role of leucine in protein metabolism during exercise and recovery. Review. *Can J Appl Physiol*. Dec 2002;27(6):646-63.
36. Beutheu S, Ouelaa W, Guerin C, et al. Glutamine supplementation, but not combined glutamine and arginine supplementation, improves gut barrier function during chemotherapy-induced intestinal mucositis in rats. *Clin Nutr*. Sep 25 2013;doi:S0261-5614(13)00241-0
37. Swaid F, Sukhotnik I, Matter I, et al. Dietary glutamine supplementation prevents mucosal injury and modulates intestinal epithelial restitution following acetic acid induced intestinal injury in rats. *Nutr Metab (Lond)*. Aug 7 2013;10(1):53. doi:1743-7075-10-53
38. Flynn NE, Shaw MH, Becker JT. Amino Acids in Health and Endocrine Function. *Adv Exp Med Biol*. 2020;1265:97-109. doi:10.1007/978-3-030-45328-2_6
39. Biezanowska-Kopec R, Leszczynska T. The effect of methionine supplementation of the AIN-93G semi-synthetic diet on the levels of homocysteine and lipids in experimental rats. *J Nutr Health Aging*. Apr 2012;16(4):395-400.
40. Amaral CL, Bueno Rde B, Burim RV, Queiroz RH, Bianchi Mde L, Antunes LM. The effects of dietary supplementation of methionine on genomic stability and p53 gene promoter methylation in rats. *Mutat Res*. May 18 2011;722(1):78-83. doi:10.1016/j.mrgentox.2011.03.006
41. Henkel AS, Elias MS, Green RM. Homocysteine supplementation attenuates the unfolded protein response in a murine nutritional model of steatohepatitis. *J Biol Chem*. Nov 13 2009;284(46):31807-16. doi:10.1074/jbc.M109.017970
42. Soderstrom E, Eliasson M, Johnson O, et al. Plasma folate, but not homocysteine, is associated with Apolipoprotein A1 levels in a non-fortified population. *Lipids Health Dis*. 2013;12:74. doi:10.1186/1476-511X-12-74
43. Yasar A, Gunduz K, Onur E, Calkan M. Serum homocysteine, vitamin B12, folic acid levels and methylenetetrahydrofolate reductase (MTHFR) gene polymorphism in vitiligo. *Dis Markers*. 2012;33(2):85-9. doi:10.3233/DMA-2012-0908
44. Nygard O, Vollset SE, Refsum H, et al. Total plasma homocysteine and cardiovascular risk profile. The Hordaland Homocysteine Study. *Jama*. Nov 15 1995;274(19):1526-33.
45. Olthof MR, Brink EJ, Katan MB, Verhoef P. Choline supplemented as phosphatidylcholine decreases fasting and postmethionine-loading plasma homocysteine concentrations in healthy men. *Am J Clin Nutr*. Jul 2005;82(1):111-7. doi:82/1/111 [pii]
46. Pavillard V, Drbal AA, Swaine DJ, Phillips RM, Double JA, Nicolaou A. Analysis of cell-cycle kinetics and sulfur amino acid metabolism in methionine-dependent tumor cell lines; the effect of homocysteine supplementation. *Biochem Pharmacol*. Apr 15 2004;67(8):1587-99. doi:10.1016/j.bcp.2004.01.006
47. Trabetti E. Homocysteine, MTHFR gene polymorphisms, and cardio-cerebrovascular risk. Research Support, Non-U.S. Gov'tReview. *J Appl Genet*. 2008;49(3):267-82. doi:10.1007/BF03195624
48. Hamlin JC, Pauly M, Melnyk S, et al. Dietary intake and plasma levels of choline and betaine in children with autism spectrum disorders. *Autism Res Treat*. 2013;2013:578429. doi:10.1155/2013/578429
49. Bruno JV, McMahon KE, Farrell PM. Lung surfactant phospholipids as related to hydration and choline status of fasted rats. *J Nutr*. Jan 1985;115(1):85-92.
50. Crivello NA, Blusztajn JK, Joseph JA, Shukitt-Hale B, Smith DE. Short-term nutritional folate deficiency in rats has a greater effect on choline and acetylcholine metabolism in the peripheral nervous system than in the brain, and this effect escalates with age. *Nutr Res*. Oct 2010;30(10):722-30. doi:10.1016/j.nutres.2010.09.008
51. Zeisel SH, da Costa KA. Choline: an essential nutrient for public health. Review. *Nutr Rev*. Nov 2009;67(11):615-23. doi:10.1111/j.1753-4887.2009.00246.x
52. Olli K, Lahtinen S, Rautonen N, Tiihonen K. Betaine reduces the expression of inflammatory adipokines caused by hypoxia in human adipocytes. *Br J Nutr*. Jan 14 2013;109(1):43-9. doi:10.1017/S0007114512000888
53. Lawson-Yuen A, Levy HL. The use of betaine in the treatment of elevated homocysteine. *Mol Genet Metab*. Jul 2006;88(3):201-7. doi:10.1016/j.ymgme.2006.02.004
54. Black MM. Effects of vitamin B12 and folate deficiency on brain development in children. Review. *Food Nutr Bull*. Jun 2008;29(2 Suppl):S126-31.
55. Fekete K, Berti C, Cetin I, Hermoso M, Koletzko BV, Decsi T. Perinatal folate supply: relevance in health outcome parameters. Research Support, Non-U.S. Gov't Review. *Matern Child Nutr*. Oct 2010;6 Suppl 2:23-38. doi:10.1111/j.1740-8709.2010.00261.x
56. Sawitzke AD, Shi H, Finco MF, et al. The effect of glucosamine and/or chondroitin sulfate on the progression of knee osteoarthritis: a report from the glucosamine/chondroitin arthritis intervention trial. *Arthritis Rheum*. Oct 2008;58(10):3183-91. doi:10.1002/art.23973
57. Biggee BA, Blinn CM, McAlindon TE, Nuite M, Silbert JE. Low levels of human serum glucosamine after ingestion of glucosamine sulphate relative to capability for peripheral effectiveness. *Ann Rheum Dis*. Feb 2006;65(2):222-6. doi:ard.2005.036368
58. Apostu D, Lucaciu O, Mester A, et al. Systemic drugs with impact on osteoarthritis. *Drug Metab Rev*. Nov 2019;51(4):498-523. doi:10.1080/03602532.2019.1687511
59. Talent JM, Gracy RW. Pilot study of oral polymeric N-acetyl-D-glucosamine as a potential treatment for patients with osteoarthritis. *Clin Ther*. Nov-Dec 1996;18(6):1184-90. doi:S0149-2918(96)80073-7

60. Salvatore S, Heuschkel R, Tomlin S, et al. A pilot study of N-acetyl glucosamine, a nutritional substrate for glycosaminoglycan synthesis, in paediatric chronic inflammatory bowel disease. *Aliment Pharmacol Ther*. Dec 2000;14(12):1567-79. doi:apt883
61. Grigorian A, Araujo L, Naidu NN, Place DJ, Choudhury B, Demetriou M. N-acetylglucosamine inhibits T-helper 1 (Th1)/T-helper 17 (Th17) cell responses and treats experimental autoimmune encephalomyelitis. Research Support, N.I.H., Extramural Research Support, Non-U.S. Gov't. *J Biol Chem*. Nov 18 2011;286(46):40133-41. doi:10.1074/jbc.M111.277814
62. Petrella RJ, DiSilvestro MD, Hildebrand C. Effects of hyaluronate sodium on pain and physical functioning in osteoarthritis of the knee: a randomized, double-blind, placebo-controlled clinical trial. *Arch Intern Med*. Feb 11 2002;162(3):292-8. doi:ioi00844
63. Ishijima M, Nakamura T, Shimizu K, et al. Intra-articular hyaluronic acid injection versus oral non-steroidal anti-inflammatory drug for the treatment of knee osteoarthritis: a multi-center, randomized, open-label, non-inferiority trial. *Arthritis Res Ther*. Jan 21 2014;16(1):R18. doi:10.1186/ar4446
64. EFSA. European Food Safety Authority. EFSA Panel on Dietetic Products, Nutrition and Allergies (NDA). Scientific Opinion on the "Rooster Combs Extract" (RCE) as a food ingredient in the context of Regulation (EC) No 258/97. *The EFSA Journal*. 2013;11(6):3260, pp:22.

Supplementary Table S2. Gene expression levels of mammary gland differentiation markers and adiponectin of MEO culture from Study 1.

	Csn2	*Csn1s1*	*Csn1s2a*	*Csn3*	*Lalba*	*Prlr*	*Adipoq*
**Control Et**	100 ± 22	100 ± 9	100 ± 27	100 ± 20	100 ± 21	100 ± 14	100 ± 35
**ALA 6 μM**	119 ± 25	122 ± 14	248 ± 28*	146 ± 39	89.0 ± 6.5	117 ± 11	144 ± 18
**PA 50 μM**	93.7 ± 9.8	125 ± 10*	202 ± 14*	163 ± 18*	82.1 ± 13.4	119 ± 14	255 ± 85
**OA 200 μM**	133 ± 28	122 ± 18	249 ± 56*	207 ± 40*	100 ± 14	126 ± 14	179 ± 27
**Control H_2O**	100 ± 8^{a}	100 ± 12	100 ± 16^{a}	100 ± 9^{a}	100 ± 15^{a}	100 ± 12^{a}	100 ± 18^{a}
**Bet 20 μM**	125 ± 14	111 ± 2	100 ± 14	103 ± 11	116 ± 7	165 ± 18^{b}	81.1 ± 25.2
**Bet 100 μM**	161 ± 23*	102 ± 10	96 ± 17	108 ± 35	132 ± 17	151 ± 8^{b}	46.4 ± 13.7*
**HA 2.4 g/L**	121 ± 11	103 ± 7	100 ± 10	114 ± 13	110 ± 16	141 ± 17	103 ± 18
**HA 10 mg/L**	120 ± 6	101 ± 7	51.8 ± 9.4	71.1 ± 17.9	116 ± 20	149 ± 13*	76.9 ± 22.1
**Hcy 10 μM**	117 ± 9^{a}	97.6 ± 7.5	121 ± 9	19.2 ± 10.0^{b}	106 ± 9^{a}	145 ± 15^{a,b}	35.3 ± 13.3^{b}
**Hcy 50 μM**	191 ± 27^{b}	131 ± 15	153 ± 27	306 ± 62^{c}	169 ± 26^{b}	209 ± 37^{b}	73.8 ± 14.3^{a,b}
**Leu 125 μM**	129 ± 9*	92.6 ± 7.3	102 ± 9	123 ± 19	97.5 ± 4.7^{a}	144 ± 22^{a,b}	84.1 ± 16.5
**Leu 625 μM**	132 ± 23	111 ± 10	105 ± 22	138 ± 24	156 ± 16^{b}	170 ± 19^{b}	71.4 ± 15.2
**NAG 7.5 mM**	142 ± 10*	118 ± 6	117 ± 9	110 ± 9	133 ± 7	178 ± 8*	76.5 ± 12.3

Data are mean ± S.E.M. (n=5-6). Results are expressed relative to the gene expression value of controls for each compound. The effect of each treatment, at both the low and high dose, against its respective vehicle-treated control group (contrast analysis) was assessed. Data not sharing common letters indicated statistically significant differences by one-way ANOVA followed by LSD *posthoc* test. * different compared to the controls by Student's t test. Significance was set at $p<0.05$.

Abbreviations:

<u>Genes</u>: Csn2, beta-casein; Csn1s1, alpha casein 1, Csn1s2a, alpha-casein 2; Csn3; kappa-casein; Lalba, lactoalbumin; Prlr, prolactin receptor; Adipoq, adiponectin.

<u>Treatments</u>: Control Et, control wells treated with ethanol 100%; ALA, alpha linolenic acid; PA, propionic acid; OA, oleic acid; Control H_2O, control wells treated with water; Bet, betaine; HA, hyaluronic acid; Hcy, homocysteine; Leu, leucine; and NAG, N-acetyl-glucosamine.

Supplementary Table S3. Bivariate Pearson correlations between miRNAs and mRNA levels of genes related to lipid metabolism from Study 4. The value of p and R is indicated.

Correlation Matrix

		26a	27a	29a	103	200a	200b	221	222	srebp1	fasn	scd1	fto	cpt1a	cpt1b
26a	Pearson's r	—													
	df	—													
	p-value	—													
	N	—													
27a	Pearson's r	0.681 ***	—												
	df	39	—												
	p-value	<.001	—												
	N	41	—												
29a	Pearson's r	0.739 ***	0.928 ***	—											
	df	40	39	—											
	p-value	<.001	<.001	—											
	N	42	41	—											
103	Pearson's r	0.644 ***	0.616 ***	0.609 ***	—										
	df	40	39	40	—										
	p-value	<.001	<.001	<.001	—										
	N	42	41	42	—										
200a	Pearson's r	0.658 ***	0.551 ***	0.567 ***	0.754 ***	—									
	df	40	39	40	40	—									
	p-value	<.001	<.001	<.001	<.001	—									
	N	42	41	42	42	—									
200b	Pearson's r	0.536 ***	0.453 **	0.476 **	0.593 ***	0.860 ***	—								
	df	40	39	40	40	40	—								
	p-value	<.001	0.003	0.001	<.001	<.001	—								
	N	42	41	42	42	42	—								
221	Pearson's r	0.660 ***	0.753 ***	0.720 ***	0.792 ***	0.450 **	0.317	—							
	df	35	34	35	35	35	35	—							
	p-value	<.001	<.001	<.001	<.001	0.005	0.056	—							
	N	37	36	37	37	37	37	—							
222	Pearson's r	0.763 ***	0.854 ***	0.820 ***	0.795 ***	0.629 ***	0.494 ***	0.884 ***	—						
	df	40	39	40	40	40	40	35	—						
	p-value	<.001	<.001	<.001	<.001	<.001	<.001	<.001	—						
	N	42	41	42	42	42	42	37	—						
srebp1	Pearson's r	-0.182	-0.309	-0.275	-0.229	-0.103	0.132	-0.392 *	-0.348 *	—					
	df	36	35	36	36	36	36	33	36	—					
	p-value	0.274	0.063	0.094	0.167	0.538	0.431	0.020	0.032	—					
	N	38	37	38	38	38	38	35	38	—					
fasn	Pearson's r	-0.208	-0.331 *	-0.416 **	-0.350 *	-0.392 *	-0.322 *	-0.340 *	-0.353 *	0.568 ***	—				
	df	38	37	38	38	38	38	35	38	36	—				
	p-value	0.199	0.040	0.008	0.027	0.012	0.043	0.039	0.026	<.001	—				
	N	40	39	40	40	40	40	37	40	38	—				
scd1	Pearson's r	-0.361 *	-0.524 ***	-0.488 **	-0.432 **	-0.468 **	-0.381 *	-0.473 **	-0.393 *	0.439 **	0.724 ***	—			
	df	38	37	38	38	38	38	35	38	36	38	—			
	p-value	0.022	<.001	0.001	0.005	0.002	0.015	0.003	0.012	0.006	<.001	—			
	N	40	39	40	40	40	40	37	40	38	40	—			
fto	Pearson's r	-0.080	-0.360 *	-0.264	-0.316 *	-0.149	-0.007	-0.370 *	-0.407 **	0.515 ***	0.466 **	0.367 *	—		
	df	38	37	38	38	38	38	35	38	36	38	38	—		
	p-value	0.621	0.024	0.100	0.047	0.358	0.966	0.024	0.009	<.001	0.002	0.020	—		
	N	40	39	40	40	40	40	37	40	38	40	40	—		
cpt1a	Pearson's r	0.308	0.460 **	0.415 **	0.245	0.405 **	0.288	0.336 *	0.292	-0.342 *	-0.272	-0.409 **	-0.045	—	
	df	38	37	38	38	38	38	35	38	36	38	38	38	—	
	p-value	0.053	0.003	0.008	0.127	0.010	0.072	0.042	0.068	0.036	0.089	0.009	0.783	—	
	N	40	39	40	40	40	40	37	40	38	40	40	40	—	
cpt1b	Pearson's r	-0.476 **	-0.723 ***	-0.676 ***	-0.344 *	-0.289	-0.173	-0.442 **	-0.477 **	0.223	0.426 **	0.304	0.180	-0.332 *	—
	df	36	35	36	36	36	36	33	36	35	36	36	36	36	—
	p-value	0.003	<.001	<.001	0.035	0.079	0.299	0.008	0.002	0.184	0.008	0.064	0.281	0.042	—
	N	38	37	38	38	38	38	35	38	37	38	38	38	38	—

Note. * $p < .05$, ** $p < .01$, *** $p < .001$
